# Relationship between serum inhibitory activity for IgE and efficacy of Artemisia pollen subcutaneous immunotherapy for allergic rhinitis: a preliminary self-controlled study

**DOI:** 10.1186/s13223-020-0416-4

**Published:** 2020-03-04

**Authors:** Wenping Wang, Jinshu Yin, Xueyan Wang, Tingting Ma, Tianfei Lan, Qingkun Song, Yifan Guo

**Affiliations:** 1 Department of Otolaryngology, Head and Neck Surgery, Peking University Ninth School of Clinical Medicine, Beijing, China; 2 Department of Otolaryngology, Head and Neck Surgery, Capital Medical University Affiliated Beijing Shijitan Hospital, Beijing, China; 3 Department of Allergy, Capital Medical University Affiliated Beijing Shijitan Hospital, Beijing, China; 4 Department of Science and Technology, Capital Medical University Affiliated Beijing Shijitan Hospital, Beijing, China; 5 Department of General Surgery, Peking University Ninth School of Clinical Medicine, Beijing, China

**Keywords:** Allergic rhinitis, Artemisia, Subcutaneous immunotherapy, Enzyme-linked immunosorbent facilitated antigen binding, Serum inhibitory activity for IgE

## Abstract

**Background:**

Biomarkers of clinical efficacy for subcutaneous immunotherapy (SCIT) on allergic rhinitis (AR) have not been identified yet. This study aims to assess the clinical relevance of serum inhibitory activity for IgE by the method of enzyme-linked immunosorbent facilitated antigen binding (ELIFAB) during SCIT for Artemisia-sensitized AR patients.

**Methods:**

19 AR patients were studied who had undergone Artemisia-specific SCIT for more than 8 months (19.68 months on average, ranging from 9 to 33 months). Peripheral bloods were collected before and after treatment. The serum inhibitory activity for IgE was tested by ELIFAB and the level of Artemisia-specific IgG4 (Artemisia-sIgG4) was determined by ELISA. Clinical improvement was evaluated based on the symptom scores and rescue medication use (SMS). The 2-tailed Wilcoxon signed-rank test and the Spearman rank test (two-tailed) were used to analyze data by using SPSS 20.0, with P values of less than 0.05 considered as significant.

**Results:**

The SMS decreased significantly after SCIT (before: 12.79 ± 4.250, after: 6.11 ± 3.828, P = 0.000 < 0.01), the treatment was remarkably effective for 6 patients, effective for 10 and ineffective for 3, along with a total effective rate 84.21%. The serum inhibitory activity for IgE increased significantly after SCIT (P < 0.05) and was correlated with the levels of Artemisia-sIgG4 (r = − 0.501, P = 0.002 < 0.01). The levels of Artemisia-sIgG4 elevated dramatically after treatment (P < 0.01) and were related with the duration of treatment (r = 0.558, P = 0.000 < 0.01). But there was no relationship between clinical improvements and the serum inhibitory activity for IgE.

**Conclusions:**

The serum inhibitory activity for IgE increased significantly after SCIT, however, there was no correlation between it and clinical improvements by statistics analysis. So whether the serum inhibitory activity for IgE can act as biomarker of efficacy for SCIT or not needs to be studied further.

## Background

Allergic rhinitis (AR) is an inflammatory disease of the nasal mucosa, induced by an IgE-mediated reaction in atopic subjects [1]. In the past decade, the prevalence of AR in China has increased to 17.6% [2] and AR has become an important issue affecting public health. Allergen immunotherapy (AIT) is the only disease-modifying treatment option available for patients with IgE-mediated allergic diseases [3] and is recommended to treat AR in severe cases [4], the clinical efficacy of which have been proven by numerous clinical trials and meta-analysis [5–8]. The success of AIT involves in many mechanisms, including the inhibition for IgE-mediated responses. As a part of it, the inhibition of binding of IgE–allergen complexes to B cells can be tested by the IgE-FAB assay [9]. It has been demonstrated that the serum inhibitory activity for IgE, determined by the IgE-FAB assay, increased after AIT and had relevance with the clinical improvements [10, 11]. Moreover, it has been recommended as potential biomarker for efficacy of AIT in 2017 EAACI Position Paper [12]. It seems that the allergen specific IgGs, especially IgG4s, play a key role in the inhibitory activity for IgE, as the depletion of total IgGs lead to the reduction of the inhibition [11, 13] and it has close relationship with serum levels of sIgG4 [11]. Although the IgE-FAB assay is reproducible, it is complex and limited to specialized centers or laboratories. There is an available alternative test, the enzyme-linked immunosorbent-facilitated antigen binding (ELIFAB) assay [14], which can also detect the inhibitory activity for IgE. Several studies have studied serum IgE inhibition by this method, which focused on insect venom allergy [15] and wasp venom allergy [16]. But there are limited researches focused on the clinical relevance of the inhibition tested by ELIFAB.

Recently Artemisia is reported to be the most common outdoor aeroallergen in Beijing [17] so it’s essential to do researches focused on Artemisia-sensitized AR. Researchers [18] have found that Artemisia pollen contains mainly five allergenic structures. Art v1 is a glycoprotein to which 90% of individuals allergic to Artemisia have specific IgE. A 60 kDa monomeric acidic glycoprotein can be recognized by the IgE from 73% of Artemisia-allergic patients. Besides, other IgE-binding structures have been detected in Artemisia pollen with described prevalence of sensitization ranging from 30 to 50%, such as glycoprotein Art v 2, non-specific lipid transfer protein (LTP) Art v 3, and profilin Art v 4. Art v 3 is responsible for the cross-reactivity between Artemisia and Rosaceae fruits (peach, apple and so on) [19], and LTPs are considered as the potential panallergens of plant allergens [20].

## Methods

### Aim, design and setting

In this study, Artemisia-sensitized AR patients were chosen as subjects, and the main purpose was to analyze the clinical relevance of the serum inhibitory activity for IgE tested by ELIFAB assay, by detecting the serum before and after subcutaneous immunotherapy (SCIT), one of the predominant forms of AIT. It was a self-controlled study, that is, indicators were compared before and after SCIT for each individual subject.

### Subjects

Patients with AR were enrolled in the study who sought treatment at Department of Allergy, Capital Medical University affiliated Beijing Shijitan Hospital between September, 2016 and May, 2018. They should meet the following criteria: (1) be diagnosed with allergic rhinitis according to ARIA 2008 diagnostic criteria, with or without asthma [21]; (2) had positive skin prick tests (≥++) and/or sIgE (> 0.35 kU/l) to Artemisia; (3) agreed to take regular Artemisia SCIT and could continue the treatment for more than 8 months, which means that they were in the maintenance phase of SCIT. The patients who: (1) had received AIT before; (2) interrupted the SCIT by themselves; (3) got the main diagnosis with atopic diseases other than AR, should be excluded.

Before the initiation of SCIT, blood sample was collected and clinical evaluation was done for each subject. Also, when they came back to the outpatient for follow-up visit after more than 8 months SCIT, blood samples were collected and clinical evaluation were done again.

### Blood samples

Serum samples from subjects were collected before and after SCIT, immediately centrifuged at 1500 rpm, 10 min, 4 °C, and stored at − 70 °C until used for detection.

#### Skin prick test (SPT)

All the subjects enrolled in the study suffered allergic symptoms, like sneezing, rhinorrhea in late summer and autumn and were diagnosed as seasonal AR. They underwent SPT testing with extracts of four main autumn pollen allergens in Beijing area (Artemisia, Ambrosia, chenopodium, and Humulus scandens, Beijing Macro-Union Pharmaceutical Limited Corporation, Beijing, China). The subjects discontinued the antihistamine at least 72 h before SPT testing. The positive control was histamine hydrochloride (10 g/L) and the negative control was glycerine saline. After disinfecting the palmar skin of the subjects’ forearm, one drop of extract fluid, one drop of negative control fluid and one drop of positive control fluid were placed at an interval of 2 cm. The standard needle was inserted into the dermis vertically through the droplet, and was removed vertically after being maintained for 1 s. The result would be determined in 15 min.

Wheal diameter = (longest diameter of the wheal + vertical diameter through the middle of the long diameter)/2. The diameter of the wheal for positive control group should be > 3 mm, while the negative control group should have no wheal. Skin index (SI) = allergen wheal diameter/histamine wheal diameter. Skin reactivity was graded according to SI: 0 < SI < 0.5 was “+”; 0.5 ≤ SI < 1 was “++”; 1 ≤ SI < 2 was “+++”; SI ≥ 2 was “++++”. Skin reactivity to allergens ≥++ was regarded as SPT positive. It’s reported that reactivity ≥++ has the strongest screening ability for Artemisia AR [22].

### SCIT protocol

**Fig. 1:**
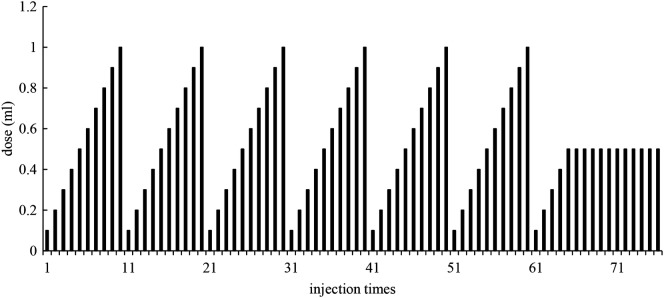
The SCIT protocol. (The concentration of extracts increased 10 times every 10 injections, until it became 1:10^2^. During the maintenance phase, the concentration was 1:10^2^ and the dose was 0.5 ml.)

The patients were treated with s.c. injections of standardized Artemisia allergen extracts (Beijing Macro-Union Pharmaceutical Limited Corporation, Beijing, China). The regular SCIT protocol included updosing phase and maintenance phase. The initial concentrations of extracts were set according to the SPT levels of subjects and the higher the level, the lower the concentration. The patients were injected with 0.1 ml extracts with the initial concentration for the first time. The dose increased by 0.1 ml for each subsequent injection and it became 1 ml for the 10th injection. Then extracts with 10 times higher concentration was used and similar process was repeated. Injections were given twice a week, extracts of each concentration could be used for 5 weeks. When the concentration became 1:10^2^, which was the highest concentration of updosing phase, the maintenance phase started. 0.5 ml of extracts was injected twice a week. After maintenance for 1 year, it could be changed to once a week as appropriate. The SCIT protocol was shown in Fig. 1. And if the adverse reaction occurred or it was during the pollen period, the concentration was appropriately lowered.

In the study, subjects were treated with multiallergen immunotherapy as most of them were polysensitized to more than one allergen. The types of allergens in SCIT corresponded to patients’ sensitization spectrum. All of them took Artemisia SCIT.

### Detection of sIgE and sIgG4

The serum levels of Artemisia specific IgE (Artemisia-sIgE) were determined in Department of Allergy, Capital Medical University affiliated Beijing Shijitan Hospital.

The concentrations of Artemisia specific IgG4 (Artemisia-sIgG4) in sera were measured by a four-layer sandwich ELISA transformed from a similar method [23]. In brief, ninety-six-well plates (467320, Nunc, Denmark) were coated overnight at 4 °C with 100 μL of allergen extracts (XP61D3A2.5, Stallergenes Greer, USA) at a 1:1000 dilution (see detail in Additional file 1). Test wells were incubated with 10 μL of the sera sample and 40 μL 1% BSA/PBS (bovine serum albumin (BSA) dissolved in phosphate-buffered saline (PBS, pH7.6; P3813, Sigma-Aldrich, Germany) at the concentration of 1%), while control wells were incubated with 50 μL 1% BSA/PBS. Higher concentration of sIgG4 in our laboratory was selected as the standard, and its concentration was set as 1 U/mL. Assay standards (diluted in 1% BSA/PBS) ranging in concentration from 0 to 0.5 U/mL were added to standard wells. After adding secondary antibody (Mouse Anti-Human IgG4 pFc, 9190-05, Southern Biotech, USA)at a 1:4000 dilution and 3,3′,5,5′-tetramethylbenzidine (TMB, PR1210, Solarbio, China), sIgG4 was determined at 450 nm using an ELISA plate reader (SPARK 10 M, Tecan, Switzerland). All standards, sera samples, and controls were tested in duplicate. The readings of tested serum should be between the minimum and the maximum readings of assay standards, otherwise they needed to be diluted and tested again. Antibody levels in the sera were quantified by extrapolation against the standard curve.

### Serum inhibitory activity for IgE [14]

In brief, 20 μl of indicator serum exhibiting high IgE concentrations for Artemisia (> 100 kU/l) was incubated with 20 μl of sample serum in the presence of Artemisia pollen extracts (XP61D3A2.5, Stallergenes Greer, USA) for 1 h at 37 °C, allowing allergen-IgE complex formation. Adding 20 μl of RPMI 1640 medium (SH30809.01, Hyclone, USA) instead of sample serum served as control. For complex formation the optimal antibody/allergen ratio was found at concentrations of 1:500 by applying the appropriate indicator serum (see detail in Additional file 1). Allergen-IgE complexes were transferred to microtiter plates coated with soluble CD23 protein (123-FE-050, R&D systems, USA) and incubated for 1 h at room temperature. After addition of biotin-conjugated anti-human IgE antibody (555858, BD Biosciences, Germany), streptavidin-peroxidase (E2886, Sigma-Aldrich, Germany), and 3,3′,5,5′-tetramethylbenzidine (PR1210, Solarbio, China), allergen-IgE complexes bound to immobilized CD23 were determined at 450 nm using the microplate reader. All samples were measured in duplicate. Data were expressed as binding of allergen-IgE complexes relative to the binding with indicator serum alone, calculated as:

$${{{\text{OD45}}0_{\text{tested}} } \mathord{\left/ {\vphantom {{{\text{OD45}}0_{\text{tested}} } {{\text{OD45}}0_{\text{indicator}} }}} \right. \kern-0pt} {{\text{OD45}}0_{\text{indicator}} }}\, \times \, 100\% .$$

### Efficacy evaluation

The effectiveness of SCIT was based on the improvement of clinical symptoms and the reduction of concomitant drugs. AR symptoms were assessed using the rhinoconjunctivitis total symptom score (RTSS). It included the symptoms of nasal discharge (rhinorrhea), nasal congestion, itchy nose, sneezing, ocular pruritus, and watery eyes, each with a four-point scale: 0 = no symptoms, 1 = mild symptoms, 2 = moderate symptoms, and 3 = severe symptoms, resulting in a possible total score of 0 to 18. As the symptoms could not be completely alleviated by the AIT treatment, especially when the allergen load was heavy, some rescue medication would be prescribed. The rescue medication score was assessed by the following standards: (1) point for oral or intranasal antihistamines, (2) points for nasal corticosteroids and (3) points for oral corticosteroids. Combined symptom medication scores (SMS) were defined as the sum of RTSS and rescue medication scores [24].

Subjects were asked to assess the severity of their allergic rhinitis symptoms and the usage of rescue medications at baseline (SMS_before_) and after SCIT (SMS_after_) according to aforementioned questionnaire.

Clinical improvement was calculated as: $\Delta {\text{SMS}}\, = \,{{\left( {{\text{SMS}}_{\text{before}} - {\text{SMS}}_{\text{after}} } \right)\, \times \, 100\% } \mathord{\left/ {\vphantom {{\left( {{\text{SMS}}_{\text{before}} - {\text{SMS}}_{\text{after}} } \right)\, \times \, 100\% } {{\text{SMS}}_{\text{before}} }}} \right. \kern-0pt} {{\text{SMS}}_{\text{before}} }}$, which was considered as remarkably effective if larger than 65%, effective if larger than 25%, otherwise the SCIT was considered ineffective.

### Statistical analysis

The 2-tailed Wilcoxon signed-rank test was used for within-group comparisons. Correlations were assessed by the Spearman rank test (two-tailed). Analyses were performed by using SPSS 20.0 with P values of less than 0.05 considered as significant, and pictures were created by using Prism software (GraphPad Software, USA).

## Results

### Subjects characteristics

19 subjects were finally enrolled in the study and their basic information is showed in Table 1. Most of subjects were sensitized to more than 1 pollen allergens so Table 2 shows their sensitization to several common autumn pollen allergens.

**Table 1:** Basic information of subjects

Items	Values
Sex(male/female)	10/9
Age(y), mean ± SD	44 ± 16.27
Duration lengths(m), mean(range)	19.68 (9–33)
sIgE (KU/L), mean ± SD	20.89 ± 19.09
SPT, level(patients number)	2(2)/3(5)/4(6)/> 4(6)

**Table 2:** Sensitization to several common autumn pollen allergens of subjects

Sensitization^a^	Allergen types			
	Artemisia	Ambrosia	Chenopodium	Humulus scandens
Patients				
A	+	+	+	+
B	+	–	+	+
C	+	–	/	/
D	+	+	/	/
E	+	+	–	/
F	+	–	–	+
G	+	+	–	–
H	+	–	/	/
I	+	–	/	/
J	+	+	+	+
K	+	+	–	/
L	+	+	+	+
M	+	+	+	/
N	+	/	/	/
O	+	–	–	+
P	+	+	+	/
Q	+	–	/	/
R	+	–	–	–
S	+	+	/	/

^a^+: positive; −: negative; /: unmeasured

### Main results

**Fig. 2:**
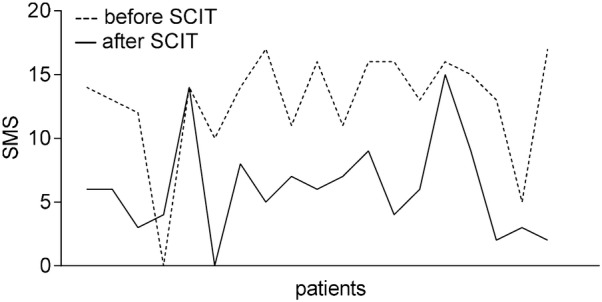
Changes of SMS during SCIT

**Fig. 3:**
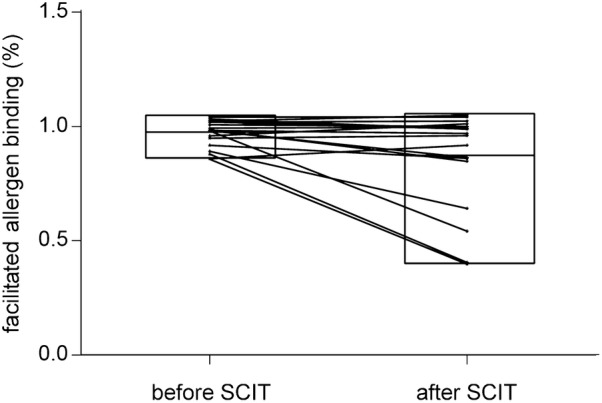
Changes of the facilitated allergen binding during SCIT (In this figure, the upper bound of the bar is the maximum, the lower is the minimum, and the middle line is the mean.)

**Fig. 4:**
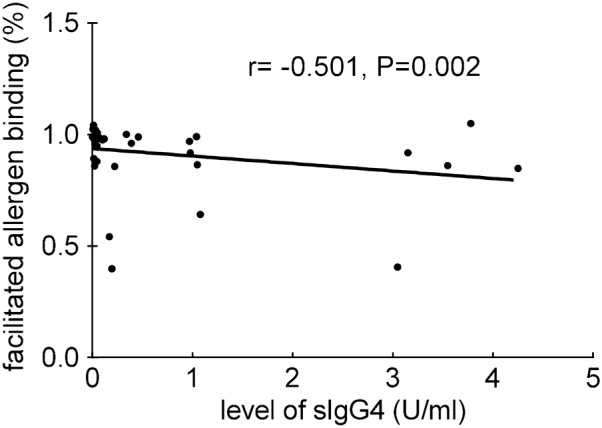
Relationship between facilitated allergen binding and serum levels of sIgG4

**Fig. 5:**
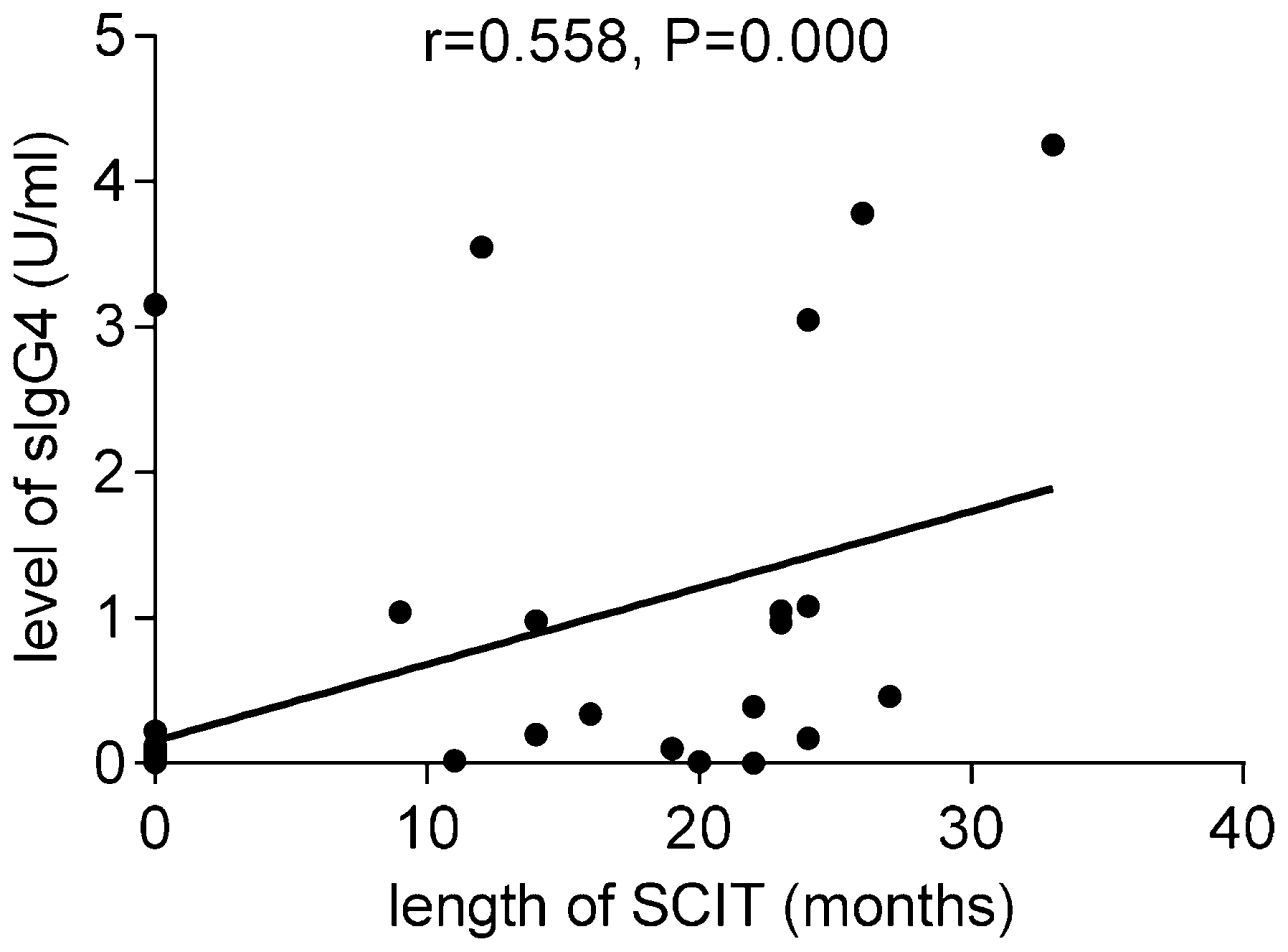
Relationship between serum levels of sIgG4 and lengths of SCIT

As showed in Table 3, SMS decreased significantly after SCIT (Fig. 2), according to the criteria for clinical improvements, patients who got remarkably effective improvements were 6, effective were 10 and ineffective were 3, along with a total effective rate 84.21%. The facilitated allergen binding decreased significantly after SCIT (Fig. 3), 15 patients had a decrease in facilitated allergen binding, 7 of which had a slight decrease. And the serum inhibitory activity for IgE increased significantly after immunotherapy. The sera level of Artemisia-sIgG4 elevated dramatically after SCIT (P < 0.01). The correlation index between serum Artemisia-sIgG4 level and facilitated allergen binding is r = -0.501 (F = 35, P = 0.002 < 0.01) (Fig. 4). The serum level of Ar-sIgG4 related significantly with duration of SCIT (r = 0.558, F = 35, P = 0.000 < 0.01) (Fig. 5), however, had no relationship with clinical improvements.

**Table 3:** Change of indicators during treatment

Indicators	Before SCIT	After SCIT	Significance
SMS (mean ± SD)	12.79 ± 4.250	6.11 ± 3.828	P = 0.000 < 0.01
Facilitated allergen binding(mean ± SD)	0.9691 ± 0.0602	0.8685 ± 0.2110	P < 0.05
Artemisia-sIgG4 (U/ml) (mean ± SD)	0.221 ± 0.733	1.129 ± 1.411	P < 0.01

The relationship between change of serum inhibitory for IgE during SCIT and clinical improvements, between clinical improvements and serum inhibitory for IgE after SCIT, between SMS_after_ and serum inhibitory activity for IgE after SCIT, between SMS_after_ and change of serum inhibitory for IgE were analyzed. However, no significant relationship between serum inhibitory for IgE and clinical improvements was found (P > 0.05 for all).

## Discussion

We found that SCIT can effectively relieve allergic symptoms and reduce the use of rescue drugs (P < 0.05), resulting total effective rate as 84.21%. Artemisia-sIgG4 increased significantly after SCIT, and had a moderate correlation with treatment duration (r = 0.558, P < 0.01), consistent with former studies which reported allergen specific IgG4s could increase in time-dependent manner [10, 25] during AIT. Allergen specific IgG4s are bispecific antibodies that may block antigenic epitopes and act as blocking antibodies and it may be responsible for postimmunotherapy serum inhibitory for IgE [12]. By ELIFAB, it was found that serum inhibitory activity for IgE increased significantly after SCIT (P < 0.05) and correlated moderately with levels of AR-sIgG4 (r = − 0.501,P < 0.01), as in former studies [10, 11]. However, there is no significant relationship between serum inhibitory activity for IgE and clinical improvements, which is different from what have been reported.

IgG4s is specific, for it has two different antigen-combining sites, so-called bispecific activity, so it can’t clear allergens efficiently as other immunoglobulins but maybe more effective as an inhibitor of allergen presentation, because it reduces complex size and hence reduces B cell activation [26]. Besides, AIT can induce the production of IgG to conformational epitopes, just like IgE, which are likely to reduce the IgE binding to allergen, thereby preventing symptoms [27]. Experiment results also proved that allergen specific IgG4s had close relationship with serum inhibitory activity for IgE, including IgE-FAB and IgE-Blocking Factor (IgE-BF) [25]. But it seems that not all allergen sIgG4s inhibit IgE binding to allergens, the epitope specificity and affinity of IgGs but not their isotype are decisive for their protective activity [28]. Maybe this could explain to certain extent why serum levels of allergen sIgG4s had no relationship with the clinical benefits [10, 25, 29]. Further, its bispecific activity makes it possible that allergen sIgG4 could oligomerize allergen molecules. Consistently, Eckl-Dorna et al. mentioned that allergen specific IgG could further oligomerize IgE-allergen complexes by super-crosslinking, leading to the crosslinking of IgE^+^ BCRs (B cell receptors) and thus activation of effective T cells, as well as the mediator release of mast cells (MCs) [30]. So the sIgGs, especially sIgG4s, can not only act as inhibitory antibodies to serve as protective factor, but exacerbate the allergic inflammation.

In the context of allergy, activation of allergic-specific T cells is the key step to induce allergic symptom. Especially, T cell activation mediates late phase reactions by increasing the levels of Th2 cytokines and then recruiting eosinophil and causing tissue damage and remodeling [31]. Receptor-mediated internalization of allergen-IgE complexes via high (FcεRI) affinity and low (CD23) affinity receptors for IgE by APCs-a process called facilitated antigen presentation (FAP)—has been shown to stimulate allergen-specific T cell proliferation more efficiently, in particular at low concentrations of allergen as they occur in vivo in allergic patients, and CD23-mediated FAP by non-cognate B cells is an important mechanism in driving AR [30]. The process FAP is tested in ELIFAB and it was indeed inhibited after SCIT in this study and other former reports [15, 16, 32], but there are many other ways to activate T cell and further induce T cell proliferation and cytokine production. T cells can be activated by internalization of allergen via fluid phase endocytosis by APCs [30]. Further, it’s the degranulation of mast cells and basophils that leads to allergic inflammation and symptoms, like nasal congestion, itchy nose and ocular pruritus directly, but activity of mast cells and basophils is influenced by many factors besides activated T cells. It was found that serum sIgE/tIgE ratio may determine the density of FcɛRI-bound allergen sIgE and therefore the likelihood of allergens to cross-link FcɛRI and to induce basophil activation in allergic subjects [33]. MCs activation are mainly mediated by cross-linking of allergen-sIgE complexes and FcεRI receptors on the membrane surface of MCs [34], in some cases allergen specific IgGs can crosslink FcεRI bound allergen-IgE complexes and aggravate allergic inflammation [30].

In addition, CD23 can bind to not only allergen-IgE complexes, but also free IgE and IgE-allergen complexes of different size and composition [35], which can disturb the detection of allergen-IgE complexes.

It was concluded that Artemisia pollen was the most allergenic pollen in northern part area of Yangtze River in China (with skin test or sIgE blood test) in 2015 [36] and an epidemiological study in 2018 [37] also found that Artemisia pollen was the most common allergenic pollen for AR patients in grasslands of northern China, implying that it’s of vital importance to study Artemisia sensitized AR in terms of treatment, prevention and so on. Furthermore, Artemisia allergy tends to be severe type [22], and almost half of the patients with autumnal pollen allergic rhinitis develop seasonal allergic asthma within 9 years [36]. Therefore, it has great clinical significance to find biomarkers which can predict or monitor the clinical efficacy of SCIT for AR patients.

There are also some limitations in this study. First, only one subject was definitely mono-sensitized to Artemisia (Table 2) and all the subjects received immunotherapy for multiple allergens at the same time, which means that their symptoms could be influenced by various kind of aeroallergens so the SMS may not be Artemisia-specific. Although 10/19 of the subjects were sensitized to both Ambrosia and Artemisia, researchers have found that patients showing poly-sensitization to both ragweed and mugwort are co-sensitized (parallel sensitization to distinct allergens) [38]. Although Ambrosia and Artemisia belong to the same plant family (the Asteraceae family) and share a number of cross-reactive allergens, their major allergens are unrelated proteins [39]. Through microarray profiling, it was found that there exists extensive cross-reactivity between them mainly involving the pan-allergens profilin and nonspecific LTPs [40]. In 2010, researchers found a novel Ambrosia allergen, Amb a 4, which contains a defensin-like domain with a high homology to Art v 1, and found IgE reaction to Amb a 4 could be inhibited by Art v 1 [41]. However, they differ in their immunological properties [42]. Amb a 1 and Art v 1, the major allergens of Ambrosia and Artemisia, respectively, are unrelated proteins. Amb a 1 is an acidic nonglycosylated protein, Art v 1 is a basic glycoprotein, they don’t cross react with each other [38]. So, the sensitization to Ambrosia may not influence the effect of Artemisia-specific immunotherapy.

Secondly, the allergen extracts used in detection of sIgG4 and ELIFAB is not the same as the extracts used in SCIT (extracts of Artemisia Tridentata and Artemisia sieversiana, respectively). It was proven by gel electrophoresis that the protein patterns of Artemisia species pollen extracts were similar, with a major band at 24 kDa, like Art v 1 [43], and it was reported that common allergen components exist in all kinds of Artemisia pollen [22], so the results may be comparable. Thirdly, the pollen levels could influence patients’ manifestations, however, the clinical evaluation and blood collection didn’t take into account the effects of pollen season and were only done at the beginning and end of SCIT, which might affect the credibility of experimental results. It would be better if we can get the pollen data for the study time frame. Then, the study design would have been more reasonable and the results would have been more powerful if it had set a placebo control group. Last, the sample is small. As we all know, there is a high dropout rate for immunotherapy [44] because of the lengthy duration and huge cost of money and time, and not all of the AR patients are willing to take SCIT because they can also get relief by taking medicines. So it’s difficult to collect subjects.

In this paper, we investigated serum inhibitory for IgE as efficacy biomarker for SCIT. However, this study was a preliminary study with a small, poly-sensitized population; therefore, a randomized, double-blind, placebo-controlled study of a large, mono-sensitized population will be needed to evaluate serum inhibitory for IgE as efficacy biomarker for SCIT.

## Conclusions

Collectively, it was found that serum inhibitory activity for IgE was significantly increased after SCIT and correlated well with serum allergen specific IgG4 levels. However, this change has no clinical relevance in this study, suggesting it’s controversial that serum inhibitory activity for IgE can be used as a biomarker to indicate the efficacy of SCIT, and further research is needed.

## Supplementary information

**Additional file 1.** It includes the introduction of allergen extract and the best concentration of allergen extract in ELIFAB and the pollen data of Beijing from August 1st to September 29th of each year from 2015 to 2018.

## Data Availability

All data generated or analysed during this study are included in this published article and its additional file.

## Abbreviations

AIT: Allergen immunotherapy
AR: Allergic rhinitis
BCRs: B cell receptors
BSA: Bovine serum albumin
ELIFAB: Enzyme-linked immunosorbent facilitated antigen binding
FAP: Facilitated antigen presentation
IgE-BF: IgE-Blocking Factor
LTP: lipid transfer protein
MCs: Mast cells
PBS: Phosphate-buffered saline
RTSS: Rhinoconjunctivitis total symptom score
SCIT: Subcutaneous immunotherapy
SI: Skin index
SMS: Combined symptom medication scores
SPT: Skin prick test
