# Supplementary material for: Relationship between serum inhibitory activity for IgE and efficacy of Artemisia pollen subcutaneous immunotherapy for allergic rhinitis: a preliminary self-controlled study
Source: Allergy Asthma Clin Immunol. 2020 Mar 4;16:18. doi: 10.1186/s13223-020-0416-4 (PMC7057474; doi:10.1186/s13223-020-0416-4)
Supplement: Supplementary file 1 — Additional file 1. It includes the introduction of allergen extract and the best concentration of allergen extract in ELIFAB and the pollen data of Beijing from August 1st to September 29th of each year from 2015 to 2018. [file 13223_2020_416_MOESM1_ESM.docx]

1.**About the allergen extract**

Reconstitute one vial of the allergen extract of Artemisia (XP61D3A2.5, Greer) in 2.5ml of double distilled water to make stock solution and then detect its concentration by BCA method as 9.386mg/ml.

Reconstitute the stock solution in PBS to make the needed concentration, such as 1:500 and 1:1000 in the article.

2.**The best concentration of allergen extract in ELIFAB**

The optimal antibody/allergen ratio to form complexes was found at an allergen extract concentration of 1:500.

3.**The pollen data of Beijing from August 1st to September 29th of each year from 2015 to 2018.**


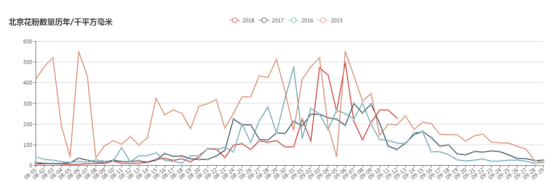


The Chinese characters “北京花粉数量历年/千平方毫米” mean that “the pollen levels of Beijing of each year/k·mm^2^”.
